# Decentralization of COVID-19 molecular diagnosis, a success story from Jordan

**DOI:** 10.7189/jogh.12.03045

**Published:** 2022-07-06

**Authors:** Arwa Qaqish, Mariam Al-Omari, Manal M Abbas, Rana Said, Mohammad Al Tamimi, Mahmoud Ghazo

**Affiliations:** 1 Department of Biology and Biotechnology, Faculty of Science, The Hashemite University, Zarqa, Jordan; 2 Department of Basic Medical Sciences, Faculty of Medicine, Yarmouk University, Irbid, Jordan; 3 Department of Medical Laboratory Sciences, Faculty of Allied Medical Sciences, Al-Ahliyya Amman; 4 Pharmacological and Diagnostic Research Lab, Al-Ahliyya Amman University, Amman, Jordan; 5 Department of Pharmacy, Faculty of Allied Medical Sciences, Al-Ahliyya Amman University, Amman; 6 Department of Basic Medical Sciences, Faculty of Medicine, The Hashemite University, Zarqa, Jordan; 7 Jordan Ministry of Health, Laboratory Directorate, Amman, Jordan

COVID-19 pandemic control measures rely on accurate and timely detection of infected individuals. Any defects and delays in diagnosis would negatively impact successful tracing and quarantining of positive cases and can significantly hinder public health control measures [1]. The World Health Organization (WHO) and the Center for Disease Control and Prevention (CDC) advocated the importance of molecular testing to control the spread of the disease. The real-time polymerase chain reaction (RT-PCR) test is considered the gold-standard method for laboratory diagnosis of COVID-19 [2]. Despite high specificity and sensitivity of RT-PCR, challenges related to a shortage in trained staff, equipment or materials, and long turnaround time (TAT) all contribute to delayed COVID-19 diagnosis [3]. Rapid antigen testing has been introduced to serve in cases of sample overloads and at point-of-care (POC) centres [4]. Moreover, SARS-CoV-2 antibody testing became necessary for assessing natural immunity as well as vaccine efficacy [5].

Molecular-based COVID-19 diagnosis at the ministry of health (MOH) in Jordan has gone through a very interesting success story, starting from only one central laboratory in the capital Amman to 17 laboratories spread out in the country to cover every governorate and travel border (**Table 1****,** **Figure 1**). This story has not been documented yet and we believe that it is worth telling due to possibly being beneficial in many ways.

**Table 1:** PCR facilities in Jordanian governorates and borders

No.	PCR Testing Facility	Governorate
1	Central Public Health Laboratory (CPHL)	Amman
2	Al Bashir Hospital	Amman
3	Prince Hamza Hospital	Amman
4	Salt Governmental Hospital	Balqaa
5	Princess Rahma Hospital	Irbid
6	Zarqa New Hospital	Zarqa
7	Al Omari Borders	Zarqa
8	Anjara CMHC	Ajloun
9	Jaber Borders	Mafraq
10	North Badia Hospital	Mafraq
11	Jerash Hospital	Jerash
12	Al Nadeem Hospital	Madaba
13	Karak Health Directorate (Karak CMHC)	Karak
14	Ghor Safi Hospital	Karak
15	Tafileh Hospital	Tafileh
16	Ma’an Hospital	Ma’an
17	Aqaba PCR Unit	Aqaba

CMHC – comprehensive medical health center

**Figure 1:**
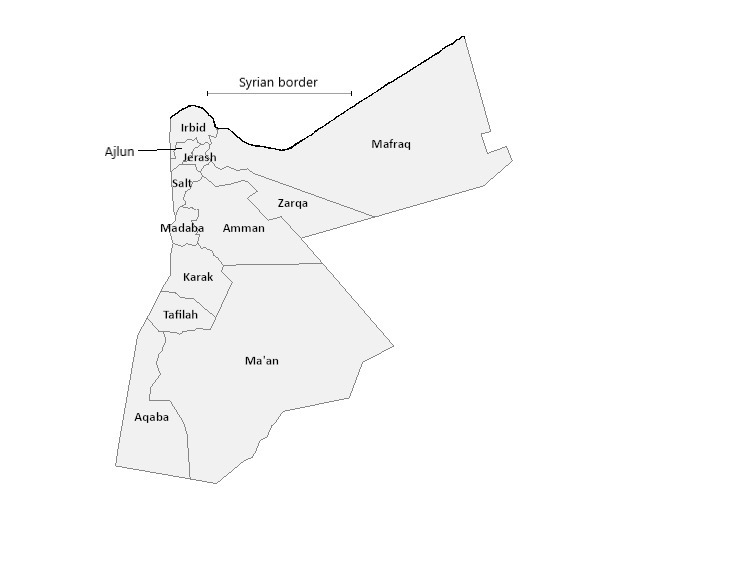
Map of Jordan showing governorates (Esri, ArcGIS online. USGS, 2019).

## DECENTRALISATION OF MOLECULAR DIAGNOSIS OF COVID-19 IN JORDAN: FROM 1 TO 17 LABORATORIES

In personal communication with Dr Mahmoud Ghazo, the Director of Laboratory Directorate at MOH, details of the COVID-19 molecular diagnosis expansion in the country were obtained and reported as follows.

Prior to COVID-19, RT-PCR testing at MOH was only available at the Central Public Health Laboratory (CPHL) in the capital of Amman. A few PCR non-real time-based genetic tests were performed at Prince Hamza molecular laboratory in Amman as well. When the pandemic hit the country with the first and second transmission waves with increasing aggressiveness, CPHL became overloaded with a huge number of samples to be analyzed from all over the kingdom, including those coming from suspected patients at hospitals and point of care centers and those collected by epidemiological investigation teams. The TAT of testing took an average of 5 to 7 days, which hindered MOH from effectively managing the pandemic (MOH, personal communication).

The fact that the private sector and university hospitals that had the capability to perform RT-PCR testing of COVID-19 would charge an average of US$40 per test in the first year of the pandemic, not covered by insurance, drew people to the MOH. The added pressure and overwhelming load posed on CPHL urged the Jordanian government to respond promptly and quickly find solution.

As part of the Jordanian government's national COVID-19 response, the MOH collaborated with private pharmaceutical and biotechnology companies to establish COVID-19 PCR testing units in all governorates and at major travel boundaries. By the beginning of 2021, 16 new COVID-19 molecular diagnosis PCR units started to operate, making sure that each governorate has its own facility (**Table 1**).

Two different private companies were involved in the establishment of governorates’ PCR units according to a tender submitted by the MOH. The site of each governorate's PCR unit was selected by the MOH, some in hospitals and others in comprehensive health centers. The private companies were responsible for providing the PCR testing units with a range of supplies and support required for their laboratories to operate with a testing capacity set by the MOH based on the estimated demand for each governorate. The MOH was responsible for supplying all PCR units, including those of university hospitals, with necessary diagnostic kits and reagents. **Table 2** lists the most used molecular assay kits in Jordan.

**Table 2:** SARS-CoV-2 PCR detection reagent kits available in Jordanian market

Kit name	Company	Target gene	Sample source	Time	Detection limit
SARS-CoV-2 Nucleic Acid Detection kit	Zybio Inc.	N gene	NS, TS, BAL stool		500 copies/m
LiliF COVID-19 Real-time RT-PCR kit	Lilif Diagnostics	RdRP genes, E and N genes	BAL, NS, OS		N/A
ViroQ® & ViroQ® Rapid SARS-CoV-2	BAG Diagnostics	E and RdRP	NP, OP, NS	90 min	5 copies/20 μl RdRP and 10 copies/20 μl target E gene
PowerChek 2019-nCoV Real-time PCR Kit	Kogenebiotech	RdRP gene, E gene	NP, OP, NS		4 copies/μL
ePlex® SARS-CoV-2 Test	GenMark Diagnostics		NPS		1x10^3^ copies/mL with WA1 viral RNA
Easy SARS-CoV- 2 WE kit	Diatech Pharmacogenetics	N and RdRp, and **and** S gene (HV69-70 del and N501Y) and the E gene	NPS, OPS, BAL	Less than 2 h	10 copies/reaction
COVID-19 Real Time Multiplex RT-PCR kit	Labsystems Diagnostics	ORF1ab, N & E besides internal control	Entire respiratory tract can be used	Approximately 1 h	<5 copies/ μl
TaqPath COVID-19 Combo Kit	Thermo Fisher Scientific	ORF1ab, N gene,S gene,MS2)	NP, OP NS, BAL		10 GCE/reaction
SNP COVID-19 REAL TIME PCR kit	SND Biotechnology	RdRp, N gene	NP, OP, ANS. nasopharyngeal wash/aspirate or nasal aspirate specimens	83 min.	between 1-10 Copies/Rxn
Yourgene Health's Clarigene SARS-CoV-2 test	Yourgene Health plc	N and E gene	NPS	1 h 20 min	5 copies per reaction
GeneFinder COVID-19 PLUS RealAmp kit	GeneFinder	RdRp gene, the E gene, the N gene, and the RNase P gene.	NPS, NS, BAL		N/A
The SARS-CoV-2 Fluorescent PCR kit	Maccura Biotechnology	ORF1ab, N and E genes	OPS, NPS, NS		1.0x103 copies/mL
VIASURE SARS-CoV-2 & UK Variant Real Time PCR Detection kit	CerTest BIOTEC	S UK, ORF1ab, and N genes	NS, NPS, OPS. Saliva		40 copies/rxn for S gene (HV 69/70 deletion), and ORF1ab gene 80 copies/rxn for N gene

NS – nasal swab, TS – throat swab, BAL – bronchoalveolar lavage, OPS – oropharyngeal swab NP – nasopharyngeal, RdRp – RNA-dependent RNA polymerase gene N. Nucleocapsid, h – hours, m – minutes

All laboratories were designed to have two physically separated rooms (extraction and PCR rooms) with a separate working station for master mix preparation. To meet the construction recommendations for a PCR laboratory, the private companies added partition walls in several sites.

In addition to participating pharmaceutical/biotechnology companies, The United States Agency for International Development (USAID) Local Health System Sustainability Project (LHSS) supported the MOH by conducting a comprehensive PCR training program for 79 MOH laboratory technicians from all governorates, including both theoretical and practical aspects. As a result, the PCR testing facility was decentralized to reach all of Jordan's geographic regions.

Along with the decentralization process, MOH developed a software system called Sundos to link all laboratories in the country performing RT-PCR diagnosis of COVID-19, including MOH laboratories, university hospitals laboratories, laboratories of military medical services, private laboratories, and laboratories at travel borders. All samples were given digital bar codes and test results were reported to the software system, allowing the MOH to track infected cases and their contacts.

## MAJOR CHALLENGE FACED BY THE DECENTRALIZATION OF COVID-19 MOLECULAR DIAGNOSIS IN JORDAN

The huge expansion in the potential of molecular diagnosis across the kingdom in a relatively short time must have been faced with many serious challenges. Regarding this issue, we interviewed directors of Jordan’s major pharmaceutical/biotechnology companies as part of our ongoing qualitative study aimed at determining the challenges in diagnosing COVID-19 in the Jordanian health sector. This study has not been published yet.

**Figure:**
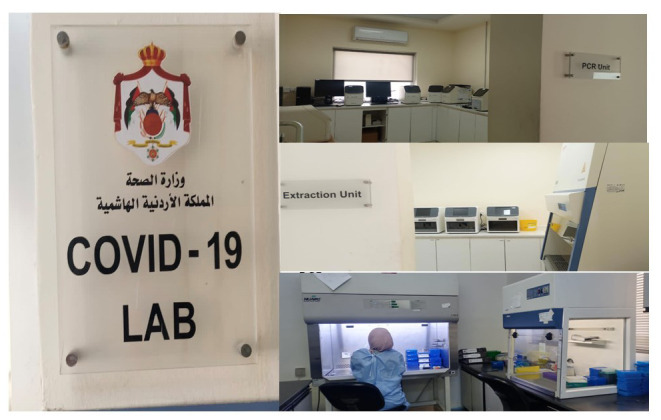
Photo: Establishment and expansion of the molecular diagnosis unit of COVID-19 in the Central Laboratory of Amman showing separate RNA extraction and PCR rooms as per the WHO standards. Source: From the authors’ own collection. Used with permission.

We identified shipping and clearance as two major challenges. Air travel restrictions and airfreight market disruption caused the delay in supplying essential health products. There has been a continuing increase in the available flights since April 2020, and thus in the capability for uniform supplies. However, there is still no indication as to when the situation is expected to return to pre-crisis levels. Furthermore, these companies faced a shortage of coronavirus test kits due to huge demand. They also confirmed taking part in designing diagnosis set ups and training laboratory specialists at Jordanian governmental and private hospitals/laboratories according to the WHO regulations based on their experience in the field. A shortage of trained staff was another challenge identified. These challenges were recently reported by a study assessing the capacity of molecular diagnosis of COVID-19 in Jordan [6].

## THE INTRODUCTION OF RAPID TESTING FOR THE DIAGNOSIS OF COVID-19 IN JORDAN

Unlike SARS-CoV and MERS, SARS-CoV-2 spread worldwide at an exceptional rate. In addition to sensitivity and specificity, the development of rapid and high through-put screening testing became a priority. From here, rapid antigen diagnostic tests (ADTs) were developed in hopes to be implemented at POC centers by frontline health care providers for detecting active COVID-19 early infection when timely admittance to molecular testing is not available [3,7]. Rapid test results should be confirmed by RT-PCR [8,9].

In 2020 and the beginning of 2021, Jordanian authorities put many restrictions on the types of commercial tests to be used through the Jordanian Food and Drug Administration (JFDA). Rapid lateral flow assays for detection of antigens were not allowed, while the use of immunoassays for antibody detection was approved. These included enzyme linked immunosorbent assay (ELISA), chemiluminescence-based platform and fluorescence-based platform with European Committee (EC), or Food and Drug Administration (FDA) quality certificates. Among the most common commercially available automated instruments used in Jordan are ARCHITECT SARS-CoV-2 N IgG Immunoassay (Abbot, Illinois, U.S.A), LIAISON® SARS-CoV-2 S1/S2 IgG (Diasorin, Saluggia, Italy), Elecsys® N Anti-SARS-CoV-2 (Roche, Mannheim, Germany), VIDAS® SARS-COV-2 RBD IgG (BioMérieux, Marcy-l'Etoile, France), Siemens SARS-CoV-2 RBD Total (COV2T) (Siemens, NY, USA), Access SARS-CoV-2 RBD IgG assay (Beckman-Coulter, CA, U.S.A.), POC instrument FREND SP IgG/IgM (Nanoentek, Korea), and Ichroma COVID-19 Ab (Ichroma /Korea).

In March 2021, the MOH, in cooperation with USAID LHSS, launched a practical training program on performing rapid ADTs for COVID-19 diagnosis. ADT use was limited to large hospitals only, including MOH hospitals and Royal Medical Services hospitals, along with the University of Jordan Hospital, King Abdullah I Hospital, the National Diabetes Center and the King Hussein Cancer Center. The JFDA only approved EC or FDA quality certified kits with sensitivity and specificity not less than 97% and 99%, respectively [10].

In February 2022, the Jordanian Pharmacists Union urged the government to allow pharmacies to perform ADTs for potential COVID-19 infections [11]. By April 30, 2022, pharmacies are still not allowed to perform or sell ADT kits.

## CONCLUSIONS

This article tells an untold story of the enormous expansion of molecular diagnosis of COVID-19 driven by MOH in Jordan. Starting from a central laboratory in the capital Amman, RT-PCR units were established in every governorate as well as travel borders to cover the whole country.

Many benefits have been achieved during the two years of pandemic. In a recent assessment of COVID-19 molecular diagnosis in the country, the number of PCR tests conducted per day significantly increased over time in correlation with the decentralization act [6]. This expansion will benefit potential future pandemics and the diagnosis of other infectious diseases and genetic conditions. Importantly, this expansion stands as a success story of cooperation between governmental and the private health sectors seeking the well-being of the country.

## References

[1] Mathuria JP Yadav R Rajkumar. Laboratory diagnosis of SARS-CoV-2 - A review of current methods. J Infect Public Health. 2020;13:901-5. 10.1016/j.jiph.2020.06.005 32534946 PMC7275982

[2] Natesan S Bhatia R Sundararajan A Dhama K Malik YS Vora K Ramping up of SARS CoV-2 testing for the diagnosis of COVID-19 to better manage the next phase of pandemic and reduce the mortality in India. Virusdisease. 2020;31:432-40. 10.1007/s13337-020-00622-x 32837973 PMC7413832

[3] Porte L Legarraga P Vollrath V Aguilera X Munita JM Araos R Evaluation of a novel antigen-based rapid detection test for the diagnosis of SARS-CoV-2 in respiratory samples. Int J Infect Dis. 2020;99:328-33. 10.1016/j.ijid.2020.05.098 32497809 PMC7263236

[4] Diao B Wen K Zhang J Chen J Han C Chen Y Accuracy of a nucleocapsid protein antigen rapid test in the diagnosis of SARS-CoV-2 infection. Clin Microbiol Infect. 2021;27:289.e1-e4. 10.1016/j.cmi.2020.09.057 33031947 PMC7534827

[5] (NIH) NIoH. Measuring protection after COVID-19 vaccination 2021. Available: https://www.nih.gov/news-events/nih-research-matters/measuring-protection-after-covid-19-vaccination. Accessed: 16 May 2022.

[6] Qaqish B Sallam M Al-Khateeb M Reisdorf E Mahafzah A Assessment of COVID-19 Molecular Testing Capacity in Jordan: A Cross-Sectional Study at the Country Level. Diagnostics (Basel). 2022;12:909. 10.3390/diagnostics12040909 35453957 PMC9024853

[7] Patel R Babady E Theel ES Storch GA Pinsky BA St George K Report from the American Society for Microbiology COVID-19 International Summit, 23 March 2020: Value of Diagnostic Testing for SARS-CoV-2/COVID-19. MBio. 2020;11:e00722-20. 10.1128/mBio.00722-20 32217609 PMC7157705

[8] Nalumansi A Lutalo T Kayiwa J Watera C Balinandi S Kiconco J Field evaluation of the performance of a SARS-CoV-2 antigen rapid diagnostic test in Uganda using nasopharyngeal samples. Int J Infect Dis. 2021;104:282-6. 10.1016/j.ijid.2020.10.073 33130198 PMC7836828

[9] Ogawa T Fukumori T Nishihara Y Sekine T Okuda N Nishimura T Another false-positive problem for a SARS-CoV-2 antigen test in Japan. J Clin Virol. 2020;131:104612. 10.1016/j.jcv.2020.104612 32871543 PMC7445490

[10] Petra. COVID-19 rapid antigen-testing training program launched. https://petra.gov.jo/Include/InnerPage.jsp?ID=32083&lang=en&name=en_news. Accessed: 29 April 2022.

[11] Times TJ. Pharmacists union calls for making rapid antigen tests available to public 2022 http://www.jordantimes.com/news/local/pharmacists-union-calls-making-rapid-antigen-tests-available-public Accesed: 30 April 2022.

